# One Step Nucleic Acid Amplification (OSNA) - a new method for lymph node staging in colorectal carcinomas

**DOI:** 10.1186/1479-5876-8-83

**Published:** 2010-09-06

**Authors:** Roland S Croner, Vera Schellerer, Helene Demund, Claus Schildberg, Thomas Papadopulos, Elisabeth Naschberger, Michael Stürzl, Klaus E Matzel, Werner Hohenberger, Anne Schlabrakowski

**Affiliations:** 1 Department of Surgery, University of Erlangen-Nuremberg, Germany; 2 Department of Pathology, Vivantes Humboldt-Clinic, Berlin, Germany; 3 Department of Molecular and Experimental Surgery, University of Erlangen-Nuremberg, Germany; 4 Department of Pathology, University of Erlangen-Nuremberg, Germany

## Abstract

**Background:**

Accurate histopathological evaluation of resected lymph nodes (LN) is essential for the reliable staging of colorectal carcinomas (CRC). With conventional sectioning and staining techniques usually only parts of the LN are examined which might lead to incorrect tumor staging. A molecular method called OSNA (One Step Nucleic Acid Amplification) may be suitable to determine the metastatic status of the complete LN and therefore improve staging.

**Methods:**

OSNA is based on a short homogenisation step and subsequent automated amplification of cytokeratin 19 (CK19) mRNA directly from the sample lysate, with result available in 30-40 minutes. In this study 184 frozen LN from 184 patients with CRC were investigated by both OSNA and histology (Haematoxylin & Eosin staining and CK19 immunohistochemistry), with half of the LN used for each method. Samples with discordant results were further analysed by RT-PCR for CK19 and carcinoembryonic antigen (CEA).

**Results:**

The concordance rate between histology and OSNA was 95.7%. Three LN were histology+/OSNA- and 5 LN histology-/OSNA+. RT-PCR supported the OSNA result in 3 discordant cases, suggesting that metastases were exclusively located in either the tissue analysed by OSNA or the tissue used for histology. If these samples were excluded the concordance was 97.2%, the sensitivity 94.9%, and the specificity 97.9%. Three patients (3%) staged as UICC I or II by routine histopathology were upstaged as LN positive by OSNA. One of these patients developed distant metastases (DMS) during follow up.

**Conclusion:**

OSNA is a new and reliable method for molecular staging of lymphatic metastases in CRC and enables the examination of whole LN. It can be applied as a rapid diagnostic tool to estimate tumour involvement in LN during the staging of CRC.

## Introduction

Carcinomas of the colon and rectum (CRC) are the fourth most common malignancy in the US and the second most common cancer for woman (195,400 cases) as well as the third most common cancer for men (217,400 cases) in Europe, with 207,400 associated deaths in 2006 [1,2]. One independent prognostic factor is the number of affected lymph nodes (LN) which are examined on the surgically resected specimen [3,4]. The number of LN which are removed during lymphadenectomy and submitted to postoperative histopathological examination can vary considerably. It is still under discussion how many LN are necessary for accurate tumor staging according to the TNM classification in CRC [5-10]. An increasing number of LN harvested by the surgeon and analysed by the pathologist has a positive influence on the patients' survival [5,11-13]. Nevertheless about 20% of initially node-negative stage UICC I and II CRC patients suffer from recurrent disease within five years after surgery [14,15]. This scenario suggests that Haematoxylin and Eosin staining (H&E) as the current method applied to assess the nodal status of CRC patients may not be fully adequate. Small metastases (<5 mm) are quite frequent in CRC patients [16]. It has been proposed that understaging in CRC is linked to the presence of occult tumour cells. Pathological investigation including immunohistochemistry (IHC) and step sectioning detected tumour deposits smaller than 2 mm in 20-30% of LN in stage UICC I and II CRC patients [15,17,18]. Molecular analysis of sentinel LN in colon carcinomas has resulted in the detection of micrometastatic disease which was undetected by IHC [19]. This is due to the fact that during RT-PCR the whole LN or at least the biggest part of the LN can be analysed while during histological work-up usually only a small part of the LN is screened. For routine purposes the workload of performing RNA extraction as a prerequisite to RT-PCR is impractical, especially when a high number of LN must be investigated. In breast cancer a molecular method called One Step Nucleic Acid Amplification (OSNA) has recently been indicated as a fast molecular diagnostic approach for the detection of LN metastases [20,21]. The aim of our study was to apply this method to LN staging in CRC patients in comparison to established histological methods.

## Materials and methods

### Patient Samples

After informed consent 184 LN from 184 patients who underwent surgery for the diagnosis of colon carcinoma were harvested. LN were selected randomised from the surgical resected specimen by the pathologist. The LN were shock frozen in liquid nitrogen immediately after surgery and stored at -80°C until further workup. The mean follow up was 72 month (range 52-88 month). Patients with a history of inflammatory bowel disease (Crohn's disease, ulcerative colitis) were excluded from the study. The study was carried out in concordance with the guidelines of the ethical commission University of Erlangen-Nuremberg and in compliance with the Declaration of Helsinki. Patients' characteristics and histopathological criteria are listed in table 1.

**Table 1:** Patients and tumour characteristics

	patients
**Age, years**	
**Mean**	66.3
**Range**	38-89
	
**Sex**	
**Male**	116
**Female**	38
	
**Grading**	
**G1**	1
**G2**	101
**G3**	70
**Gx**	12
	
**Stage; TNM 6^th ^edition**	
**IA**	10
**IB**	28
**IIA**	48
**IIB**	7
**III**	2
**IIIA**	14
**IIIB**	11
**IIIC**	23
**IV**	41
	
**Nodal status**	
**pN0**	107
**pN1**	34
**pN2**	42
**pN3**	1

### Study design

**Figure 1:**
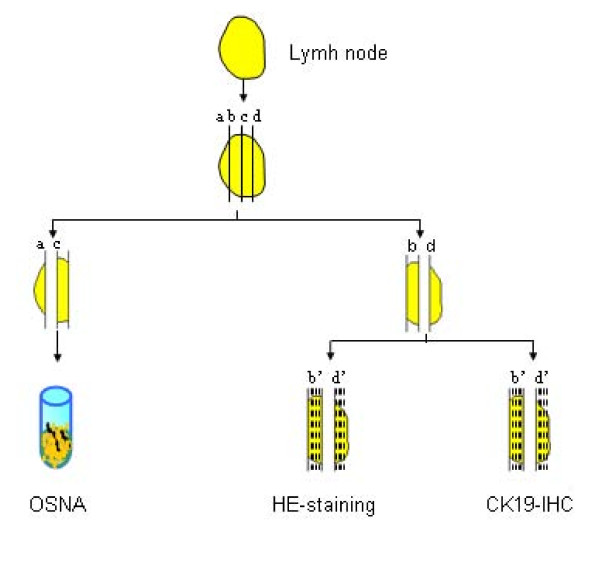
**Study design for lymph node workup**.

LN were cut into 4 slices (a, b, c, d) of each 1 or 2 mm thickness with a special cutting device or cut into halves if the LN was too small for this cutting procedure [20]. Slices a and c were analysed by OSNA, slices b and d by histology. In case discordant results were obtained, the lysates of the discordant samples were subjected to quantitative RT-PCR (qRT-PCR) with CK19 and carcinoembryonic antigen (CEA). If the result of this discordant case investigation (DCI) was supportive of the OSNA result it was concluded that the discordant result was likely to be caused by tissue allocation bias (TAB), meaning that tumor deposits were only contained in the slices designated for OSNA or in the slices used for histology. As a consequence these samples were excluded from the sample cohort because comparison of the two methods was not possible (figure 1).

### Histopathology

**Figure 2:**
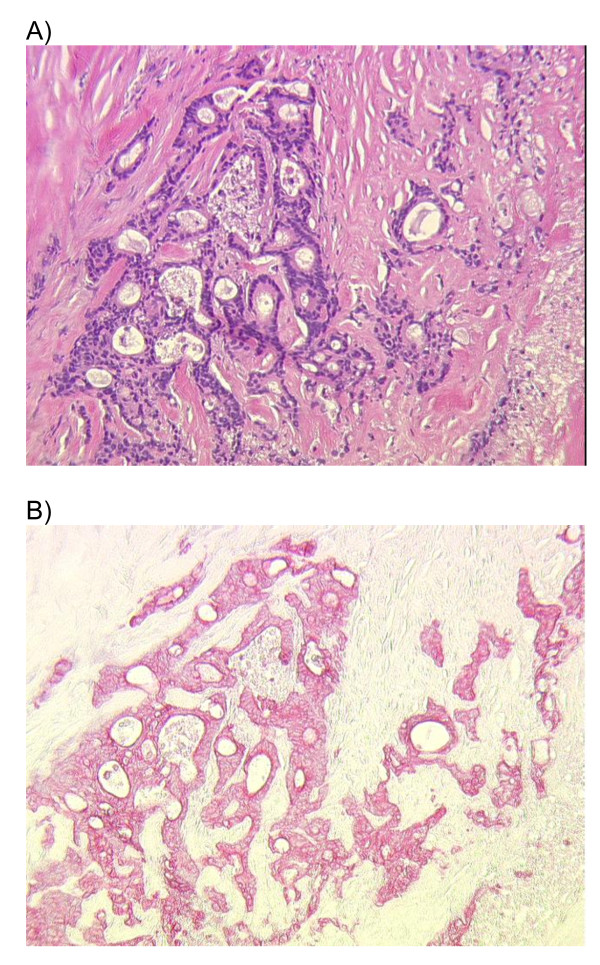
**A) H&E staining and B) CK-19 immunohistochemistry of lymph node metastases from colon carcinoma**.

5 levels of histology were performed for each slice (b, d) and results were recorded separately for each slice. Each level consisted of 2 sections, one was stained with H&E and the other one was used for CK19 IHC. Tissue was cut in 4 μm slices and dried at 60 °C. Paraffin was separated using xylol and ethanol (100%-70%). After 10 minutes of pronase digestion, the sections were incubation with a CK19 antibody (Clone M0888 and clone No. RCK 108, Dako, Glostrup, Denmark). A washing process was carried out with Tris buffer and the secondary antibody was added. Finally the staining with Fast Red TR salt (Sigma-Aldrich, Germany) completed the procedure. The background was stained with Haematoxylin (figure 2).

### One Step Nucleic Acid Amplification (OSNA)

To compare OSNA with histology, the CK19 mRNA based OSNA procedure was performed as described elsewhere [20]. CK 19 was identified as the most sensitive marker for OSNA in CRC during pilot studies (data not shown). OSNA with beta-actin was carried out as previously indicated and served as a RNA quality control [21]. The slices a and c were homogenised in 4 ml of lysing buffer for 90 seconds (Lynorhag, Sysmex, Kobe, Japan) and centrifuged for 1 minute at 10,000 g. Afterwards CK19 or beta-actin mRNA was amplified by reverse-transcription loop-mediated amplification (RT-LAMP) in the RD-100i (Lynoamp, Sysmex, Kobe) [22]. Automated amplification with a ready-to-use reagent kit (Lynoamp, Sysmex, Kobe) was performed directly from the sample homogenate, with no RNA purification necessary, according to the manufacturer's instructions. The result was released after a total of 30-40 minutes for 3-4 LN. During a pilot study in 136 LN from CRC patients without lymph node metastases less than 250 copies/μl for CK19 were evaluated as negative result for OSNA (unpublished data). Therefore results were classified as (-) for CK 19 mRNA copies/μL less than 250 (for beta-actin less than 1000 copies/μL), as (+) for CK19 mRNA between 250 - 5000 copies/μL (for beta-actin: 1000 - 5000 copies/μL), and (++) for mRNA copies/μL higher than 5000.

### Quantitative reverse-transcriptase polymerase chain reaction as part of discordant case investigation (DCI)

DCI was performed after the original analysis. OSNA runs were repeated from discordant sample homogenates and afterwards RNA was isolated and subjected to qRT-PCR for CK19, CEA, and beta-actin. Conditions for CK19, CEA and beta-actin qRT-PCR were recently reported and described elsewhere [20,21]. Primer sequences for amplification of CEA were: 5'- AGACAATCACAGTCTCTGCGGA-3' (forward) and 5'- ATCCTTGTCCTCCACGGGTT-3' (reverse). The cut-off was set at cycle time = 29.6 for CK19, 28.5 for CEA, and 30.0 for beta-actin.

## Results

### OSNA and histology

184 LN from 184 patients with colon cancer were investigated with both OSNA (CK19 mRNA as a marker) and intensive histological methods (H&E and CK19 IHC on 5 levels for each of 2 LN slices). RNA quality was assured by OSNA performed for beta-actin. 139 samples gave a negative result and 37 samples gave a positive result with both methods (table 2). No isolated tumour cells were found. In 10 out of 40 histology positive cases the metastases was only found in one slice but not in the other. Two positive samples contained a 10 mm and a 5 mm macrometastasis and OSNA (++) results, but were originally staged as IB and IIA, respectively, by routine H&E staining. This means that LN were cut in half and only one slice per of each half underwent H&E staining. Three samples were histology+/OSNA-, and 5 samples were histology-/OSNA+, accounting for a concordance rate of 95.7%, sensitivity of 92.5%, and specificity of 96.5% before DCI (table 2). Three patients (3%) underwent LN upstaging during OSNA (table 3: No 4, 5, 8) but were initially staged as LN negative during routine histopathology (table 2: stage UICC IIA, IIA, IB). During follow up one of these patients developed metachronous distant metastases (DMS) in the liver (table 4).

**Table 2:** Results of OSNA and histology (H&E Staining and CK19 IHC) for 184 LN

OSNA	HISTOLOGIC INVESTIGATION		
	**Macrometastases**	**Micrometastases**	**Negative**

**++**	27	2	2 (1)
**+**	8	-	3 (2)
**-**	3 (2)	-	139

**Total**	38 (37)	2	144 (142)

Numbers in brackets indicate samples after discordant sample analysis, ++: CK19 mRNA copies/μL higher than 5000, +:CK19 mRNA between 250 - 5000 copies/μL, -: CK 19 mRNA copies/μL less than 250

**Table 3:** Results of discordant case investigation between OSNA and histology (H&E Staining and CK19 IHC

No.	Stage UICC	Histology		OSNA				qRT-PCR			Conclusion
		**size**	**description**	**original run copies/μL**	**+/-**	**second run**^**1 **^**copies/μL**	**+/-**	**beta-actin** **+/-**	**CK19** **+/-**	**CEA** **+/-**	

1	IV	8 mm	Macrometastasis, only in slice b, not d	<250	-	5470	++	+	+	+	Discordant Sample mix-up
2	III	m	Macrometastasis, calcified tissue	<250	-	0	-	+	+	-	Discordant Beta-actin and CK19 value near the cut-off level
3	IV	3 mm	Macrometastasis, only level 2	<250	-	<250	-	+	-	-	Tissue allocation bias
4	IIA	-	negative	540	+	<250	-	+	-	-	Discordant Low copy
5	IIA	-	negative	1300	+	<250	-	+	-	-	Discordant
6	IV		negative	1300	+	290	+	+	-	+	Tissue allocation bias
7	IIIA	3.5 mm	Positive with IHC in level 1	25000	++	53700	++	+	+	+	Tissue allocation bias
8	IB	-	negative	27000	++	<250	-	+	-	-	Discordant Sample mix-up

OSNA: ++: CK19 mRNA copies/μL higher than 5000, +:CK19 mRNA between 250 - 5000 copies/μL, -: CK 19 mRNA copies/μL less than 250, ^1^The indicated copy number is the mean of three OSNA runs, QRT-PCR: +: positive, -: negative.

**Table 4:** Tumor characteristics and follow up of patients which were histopathology LN negative and underwent LN upstaging by OSNA

	Histopathology			OSNA		Follow up
**Tumor**	**T**	**N**	**M**	**N**	**DMS**	**dead/alive**

Rectum	2	0	0	1	none	alive
Rectum	3	0	0	1	Liver	alive
Sigmoid colon	3	0	0	1	none	alive

Mean follow up 72 month (range 52-88 month). DMS: distant meachronous metastases.

### Discordant case investigation (DCI)

For all discordant samples additional OSNA runs as well as qRT-PCR for CK19 and CEA were conducted from the remaining homogenate. However, since prolonged storage in the homogenising buffer may adversely affect RNA quality, data obtained by DCI may not fully reflect the original condition and this may in particular become evident in samples with CK19 mRNA copies near the cut-off level of both OSNA and DCI.

Discordant case 1 contained a macrometastasis (8 mm), but only in slice b qRT-PCR and additional OSNA runs performed from this sample during DCI yielded positive and therefore concordant results so possibly the original negative value was due to a sample mix-up. Sample 2 contained a 5 mm macrometastasis with calcified tissue. Quantitative RT-PCR was positive for CK19 only, with a cycle time close to the cut-off level, although all OSNA results for CK19 were negative. At the same time the beta-actin value was right on the cut-off level (not shown) which suggests that the RNA concentration contained in the homogenate was very low. Sample 3 contained a metastasis restricted to one out of five levels whereas both OSNA and qRT-PCR were negative, strongly indicative of tissue allocation bias (TAB), meaning that tumour deposits are restricted to the slices either used for OSNA or histology. The corresponding patient was formerly judged as pN0 by routine histopathology performed outside this study.

Three out of the 5 histology-/OSNA+ samples (4 - 6) contained CK19 mRNA copy numbers in the lower range indicative of small tumour deposits located in the slices analysed by OSNA. Two of these cases (4 and 5) were originally node-negative and further CK19 OSNA runs and qRT-PCR gave negative results. The beta-actin OSNA copy numbers for these samples were seven and four times lower at DCI, respectively, than the beta-actin values from the original run (not shown). Therefore we assume that RNA quality had suffered after a couple months of storage so that the original results could not be reproduced. In contrast to this, for sample 6, both OSNA and qRT-PCR supported the output of the original OSNA run. LN 7 exhibited (++) judgement in all OSNA runs, and IHC gave positive results in only level 1. Since this LN was rather small it was cut into 2 pieces, and the metastasis was probably for the most part located in the half used for OSNA and only with a small part present in the other half used for histology. Discordant case 8 was strongly positive in the first OSNA run but negative upon OSNA repetition and qRT-PCR so a sample mix-up cannot be excluded. In summary, 3 out of 8 discordant samples were likely to be caused by TAB. If these samples were excluded from the sample cohort, the concordance rate was 97.2% (176/181), sensitivity 94.9% (37/39), and specificity 97.9% (139/142).

## Discussion

As lymphatic metastasis is a strong prognostic indicator in CRC, the assessment of the nodal status is a key factor in CRC staging. 10-20% of node-negative stage UICC I and II patients develop systemic disease within less than 5 years. It has been suggested that using conventional histological analysis, e.g. one or several H&E sections, a certain proportion of micrometastases and disseminated tumor cells remains undetected [17,23].

In the present study, 184 frozen LN from 184 CRC patients were subjected to both, extensive histology and OSNA, with alternating slices of the LN used for each method. The comparative evaluation showed 95.7% concordance, 92.5% sensitivity, and 96.5% specificity. Lysates of the 8 discordant samples were further analysed by RT-PCR. In 3 out of 8 samples DCI supported the OSNA finding. It was concluded that metastases were strictly located in either the tissue used for OSNA or the tissue used for histology which renders a comparison of the 2 methods impossible. By excluding these samples concordance rate was 97.2%, sensitivity 94.9%, and specificity 97.9% when compared to a very extensive histological examination which is not routinely performed in all institutions. Differences between CEA and CK-19 qRT-PCR as detected in two cases reflect the heterogeneity of marker expression in lymph node metastases of CRC (table 3). These results are in concordance with previous studies which also identified a disconcordant expression between CEA and cytokeratin in lymph node metastases of CRC in about 25% [24]. In two cases there were differences between second run OSNA and qRT-PCR (table 3). In case two there was calcified tissue which may have influenced OSNA. In case six there was a low copy number of OSNA which was slightly above the positive cut off value. The low amount of CK19 mRNA in the sample was verified by qRT- PCR. Nevertheless this borderline case reflects the need of additional markers in specific cases such as CEA.

In a different study carried out with an earlier prototype of the RD-100i, 63 LN from 6 CRC patients were investigated with both H&E staining and OSNA. None of the LN was HE+/OSNA- and 3 of the 63 LN were HE-/OSNA+, resulting in upstaging of two patients [25]. The aspect of upstaging was not the main focus of this investigation since only one LN from one CRC patient was analysed. Despite this, even in the 93 LN from stage UICC I or II patients enrolled in this study tumour deposits were detected in 3 cases (3%) by OSNA. One of these patients developed hepatic DMS during follow up. Correct tumor staging is essential to apply adequate adjuvant treatment. If patients are under staged during routine histopathology they will not receive necessary adjuvant chemotherapy which may result in tumor progression during follow up. More sensitive staging methods can reduce such pitfalls and prevent tumor recurrence especially in stage UICC II. For OSNA fresh tissue is required and the lymph nodes should be harvested by pathologists from the resected specimen. This could cause a major change during clinical practise. Therefore an interdisciplinary dialogue and planning is indispensable for this procedure. Furthermore lymph node harvesting in fresh tissue is much harder compared with formalin fixed material and requires a special training. The OSNA lysate can be asservated and in unclear cases RNA isolation for further diagnostics is possible.

Our findings underline the requirement for a more comprehensive diagnostic technique than H&E staining of a limited numbers of sections has provided so far, in particular the need to analyse the whole LN in order to detect occult small tumour deposits. As opposed to a variety of different histological approaches presented so far for the identification of occult disease in LN of CRC patients the OSNA method is a standardised technique which includes a short homogenisation step and subsequent automated amplification of CK19 mRNA and therefore ensures reproducible and objective judgement [26]. In contrast to RT-PCR for which RNA purification is mandatory, in the OSNA assay amplification directly starts from the lysate and therefore allows analysis of 3-4 LN within 30-40 minutes and 12 LN within 2 hours. For the reason that increasing numbers of investigated LN correlate with a more accurate tumour staging, high throughput methods are indispensable for future purpose [5,13,14]. In conclusion, the CK19 mRNA based OSNA is a new and reliable method to determine metastatic disease in LN and can be applied as a rapid diagnostic tool during staging of CRC patients.

## Competing interests

The study was supported by the company Sysmex, Kobe, Japan. Otherwise the authors declare that they have no competing interests.

## Authors' contributions

RSC participated in the design of the study, worked up the lymph nodes, supported data workup, statistical analysis and drafted the manuscript, VS was involved in technical assistance and in writing the manuscript, HD carried out H&E staining and CK19 IHC, CS coordinated the study and drafted the manuscript, TP participated in the study design and drafted the manuscript, EN and MS added technical support and drafted the manuscript, KEM and WH participated in patient recruitment and drafted the manuscript, AS scored the H&E staining and CK19 IHC of the LN. All authors read and approved the final manuscript.
